# Fabrication of heart tubes from iPSC derived cardiomyocytes and human fibrinogen by rotating mold technology

**DOI:** 10.1038/s41598-024-64022-7

**Published:** 2024-06-07

**Authors:** Birgit Andrée, Nils Voß, Nils Kriedemann, Wiebke Triebert, Jana Teske, Mira Mertens, Merlin Witte, Sára Szádocka, Andres Hilfiker, Thomas Aper, Ina Gruh, Robert Zweigerdt

**Affiliations:** Leibniz Research Laboratories for Biotechnology and Artificial Organs (LEBAO), Department of Cardiothoracic, Transplantation and Vascular Surgery, MHH—Hannover Medical School, Carl Neuberg Str. 1, 30625 Hannover, Germany

**Keywords:** Tissue engineering, Cardiovascular diseases, Pluripotent stem cells

## Abstract

Due to its structural and functional complexity the heart imposes immense physical, physiological and electromechanical challenges on the engineering of a biological replacement. Therefore, to come closer to clinical translation, the development of a simpler biological assist device is requested. Here, we demonstrate the fabrication of tubular cardiac constructs with substantial dimensions of 6 cm in length and 11 mm in diameter by combining human induced pluripotent stem cell-derived cardiomyocytes (iPSC-CMs) and human foreskin fibroblast (hFFs) in human fibrin employing a rotating mold technology. By centrifugal forces employed in the process a cell-dense layer was generated enabling a timely functional coupling of iPSC-CMs demonstrated by a transgenic calcium sensor, rhythmic tissue contractions, and responsiveness to electrical pacing. Adjusting the degree of remodeling as a function of hFF-content and inhibition of fibrinolysis resulted in stable tissue integrity for up to 5 weeks. The rotating mold device developed in frame of this work enabled the production of tubes with clinically relevant dimensions of up to 10 cm in length and 22 mm in diameter which—in combination with advanced bioreactor technology for controlled production of functional iPSC-derivatives—paves the way towards the clinical translation of a biological cardiac assist device.

## Introduction

Cardiac tissue engineering encompasses established technologies for generating contractile bundle-, patch- or sheet-like constructs^1–3^. Recently, based on human iPSC-derived cardiomyocytes (hiPSC-CM), cardiac patches were generated and grafted onto the (left) ventricle of heart failure patients, to support the functional output of the heart in first clinical trials^4–6^(https://www.repairon.de/). However, animal experiments suggest that the functional efficiency and success of cardiac patch transplantation onto the epicardium are challenged by the limited thickness of the constructs, the lack of electromechanical coupling to the host myocardium, and the general issue of immune rejection in a xenogeneic model^6,7^. Other strategies focused on a more holistic approach of “whole organ replacement”. Proof-of-concept (PoC) studies showed for example the engineering of a single ventricle^8^, pulsatile conduits^9–13^ or even multi-chambered structures^14–16^ based on printing technologies or the re-seeding of decellularized heart matrices. Despite this progress, all previous achievements suffer from limitations regarding their clinically relevant dimensions for envisioned “whole organ replacement” or biological cardiac assist devices.

For cardiac tissue engineering several matrices including solubilized decellularized native tissues, collagen, fibrin or blends of these matrices have been employed for assembling CMs and other cell types into three dimensional (3D) constructs^2,17–20^. However, due to its abundant availability, biocompatibility, and routine use in the clinic, fibrin maybe considered as a favorable scaffold material^21^.

Although fibrin is sensitive to rapid degradation by proteolytic cells, this process can be controlled by the addition of fibrinolytic inhibitors such as aprotinin or tranexamic acid (trans-4-aminomethyl-cyclohexane-1-carboxylic acid; t-AMCA). Aprotinin is a serine protease inhibitor, specifically acting on trypsin, plasmin, and kallikreins, whereas t-AMCA competitively inhibits the activation of plasminogen to plasmin via reversible binding to the lysine-binding site on plasminogen^22^. Another challenge in the field is the achievement of sufficient biomechanical strength of fibrin-based constructs. This, however, has been addressed by the rotation-based compaction of fibrin during the process of polymerization resulting in a significant improvement of mechanical properties^23,24^. Focusing on acellular grafts, the above mentioned technique has been successfully used for the generation of artificial blood vessels including their implantation as substitutes of a segment of the carotid artery in a sheep model^24^. In this study, we have adapted the technique of a rotating mold for the fabrication of tubular cardiac constructs based on iPSC-CMs and human foreskin fibroblasts (hFFs) and revealed parameters influencing the tissue formation and remodeling of the resulting tubes.

Due to their substantial size in all three dimensions, their architecture and their physical and functional properties, these tubular cardiac grafts may serve as a potent biological assist device in future clinical applications.

## Results

### The fibrinogen quality substantially impacts the uniformity of fibrin tube formation

**Figure 1:**
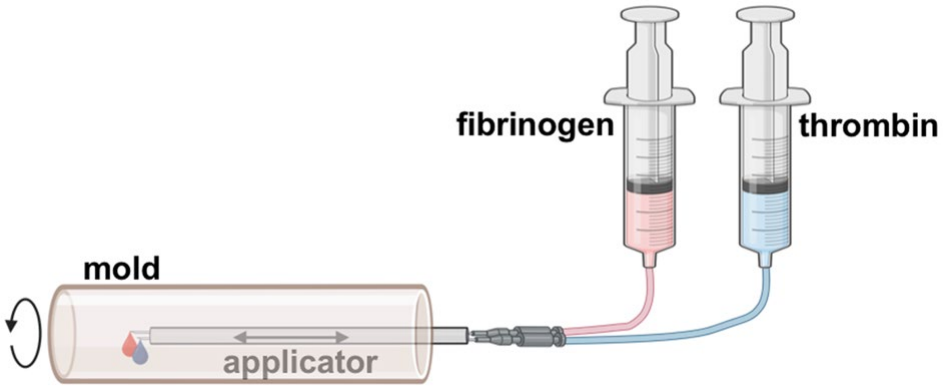
Schematic of the rotating mold technology. Two syringes containing fibrinogen solution and thrombin solution, respectively, are each connected to a drip line and a cannula. The cannulas are inserted into an applicator, which is reaching into a rotating mold and can move backward and forward. While moving, the two solutions are dispersed from the tip of the cannulas into the rotating mold. Upon contact of the two solutions the formation of fibrin fibers is initated. (Figure created with BioRender.com)

**Figure 2:**
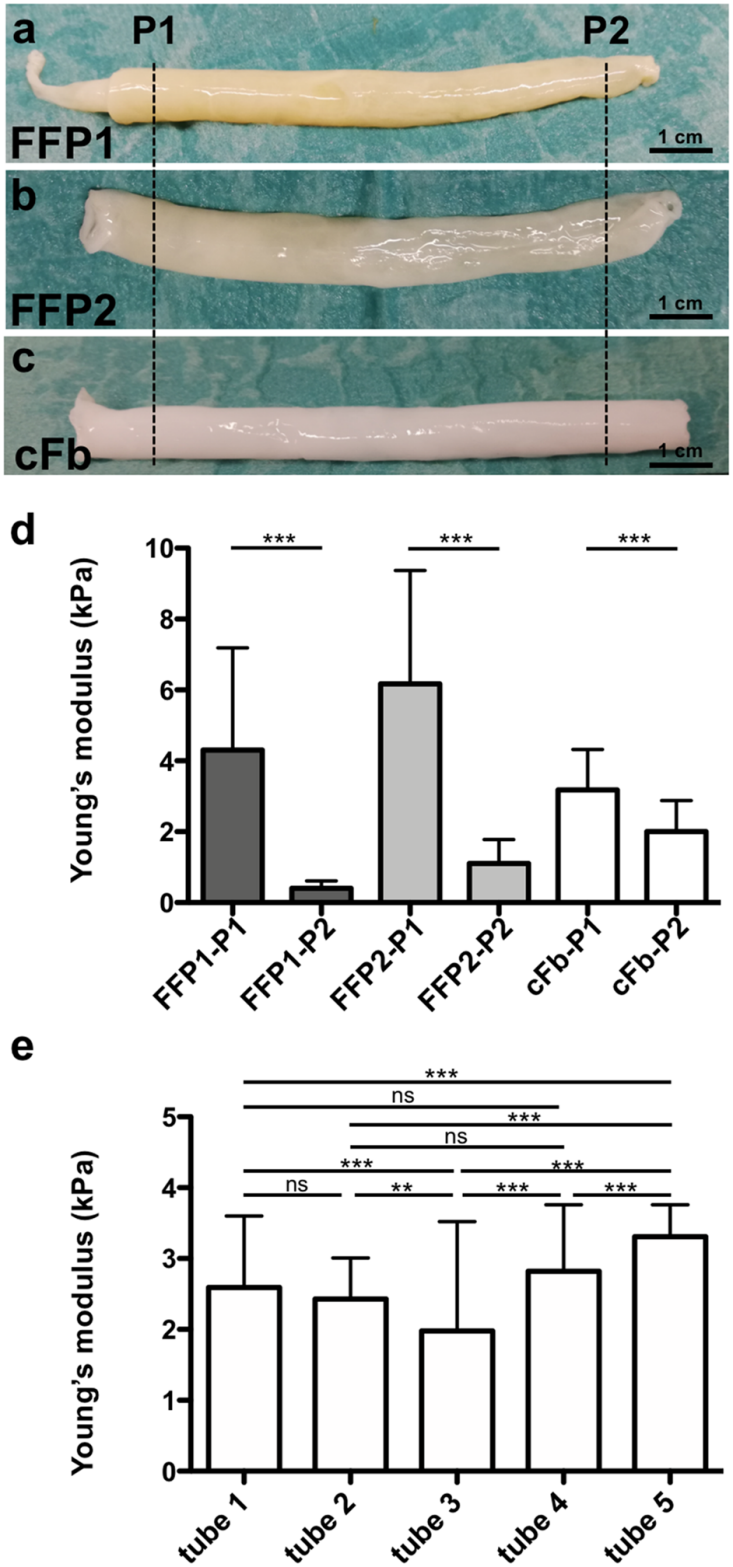
Commercially available fibrinogen improves uniformity of fibrin tubes. (**a**,**b**) Fibrin tubes fabricated with fibrinogen isolated from two independent batches of fresh frozen plasma (FFP), termed FFP1 and FFP2, respectively. (**c**) Tube produced from commercially available fibrinogen (cFb). (**d**) Young’s modulus of tubes shown in (**a**–**c**). Young’s modulus of pieces from position 1 and 2 (P1, P2) was determined by nanoindentation (3 pieces per tube were assessed by a matrix scan of 100 indentations per piece). Mean value with SD is displayed. (**e**) Young’s modulus determined by nanoindentation of five independently fabricated tubes made from cFb. 2 pieces per tube were analyzed with > 100 measurement points per piece. Mean value with SD is displayed. ns = p > 0.05, ** = p < 0.01, *** = p < 0.001. FFP: fresh frozen plasma, cFb: commercially available fibrinogen. Scale bar: 1 cm.

For the formation of fibrin-based tubes a rotating mold technology (RMT) was previously established by us^23^. In brief, for RMT two syringes filled with fibrinogen solution and thrombin solution, respectively, were each connected to a drip line and a cannula, which was inserted into an applicator (Fig. 1). The applicator is moved forward and backward along the axis of the rotating mold. By automatic control of the plungers, the solutions are dispersed from the tip of the cannulas into the rotating mold while the applicator is moving. Upon contact of the two solutions in the mold the polymerization of fibrinogen is initiated resulting in a tubular fibrin structure. Utilizing this technique, cell-free fibrin tubes were fabricated employing fibrinogen cryoprecipitated from fresh-frozen plasma (FFP). This approach, however, resulted in both macroscopically observable inhomogeneities (Fig. 2a, b) and heterogeneous mechanical properties along individual tubes as well as across independent experiments (Fig. 2d), indicating batch-dependent variations of the quantity and quality of individual FFP preparations.

Indeed, the application of commercially available human fibrinogen (cFb) promoted the production of more uniform tubes (Fig. 2c) with a similar Young’s modulus along individual tubes that is 3.2 ± 1.3 kPa found in position 1 and 2.0 ± 0.9 kPa in position 2 (Fig. 2d). Although these values are still significantly different, the increase in homogeneity is in obvious contrast to the highly variable results observed for FFP preparations, as exemplified for the tubes FFP1 (4.3 ± 2.9 kPa in position 1 versus 0.4 ± 0.2 kPa position 2) and FFP2 (6.2 ± 3.2 kPa for position 1 versus 1.1 ± 0.7 kPa for position 2) respectively (Fig. 2d). Furthermore, comparison of numerous independent cFb-derived tubes revealed a high degree of reproducibility regarding their mechanical properties with an average Young’s modulus of 2.6 ± 0.5 kPa (Fig. 2e). These findings support the notion that the use of cFb promotes the reliable fabrication of tubes with highly reproducible properties.

### A relatively high iPSC-CM density is required for achieving synchronized calcium transients in fibrin-based cardiac tubes

**Figure 3:**
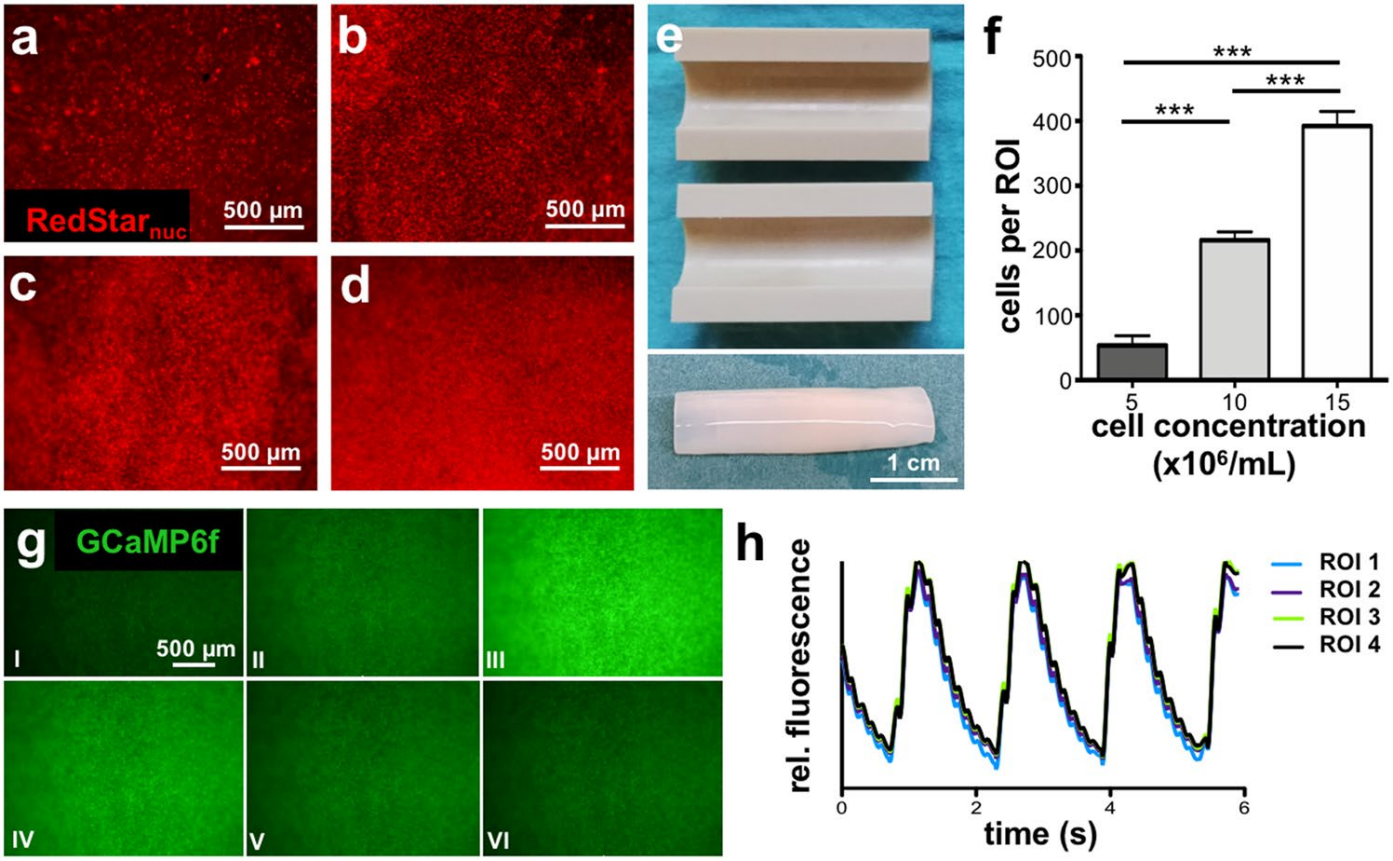
High density of iPSC-CMs leads to synchronized calcium transients. (**a–d**) Fibrin tubes fabricated with increasing cell concentrations of Ruby-derived CMs and hFFs ((a) 5 × 10^6^/mL, (b) 10 × 10^6^/mL, (c) 15 × 10^6^/mL, (d) 25 × 10^6^/mL of total cell concentration with 90% CMs and 10% hFFs) after 3 days of cultivation in serum-free media (SFM). The indicated cell concentrations refer to the cell–matrix solution applied for the tissue generation process. (**a–d**) View onto the outer tube surface revealing nuclear RedStar fluorescence. (**e**) Mold for generating cell containing fibrin tubes. Length was reduced to 3 cm. Tube from (**d**) is shown below the mold directly after the fabrication. (**f**) Quantification of cells per region of interest (ROI) in dependence of initially applied cell concentration. Four ROIs with the same dimensions were analyzed per tube. Mean and SD is depicted.*** = p < 0.001 (**g**) Still images of GCaMP6f signal recording of the tube shown in (**d**) displaying a time interval of 0.25 s between pictures I to VI demonstrating GCaMP6f activity by GFP signal intensity at day 7 of cultivation. (**h**) Calcium fluctuations represented by GFP signal intensity were analyzed over time with ImageJ employing the recording shown in supplemental movie 1. Four regions of interest (ROI) were randomly selected. Scale bar: (**a**–**d**,**g**) 500 µm, (**e**) 1 cm.

For initial experiments with cells the mold was shortened to 3 cm in length to enable efficient use of iPSC-CMs. The MHHi001-A-5 hiPSC reporter line (“Ruby”; constitutively expressing nuclear RedStar and the genetic calcium sensor GCaMP6f^25^) was applied for the generation of CMs based on our chemically defined differentiation strategy in large-scale suspension culture^26,27^. This cell line enables both, evaluation of final cell density and monitoring of electrical coupling of hiPSC-CMs in living tissues. As the cell density is a crucial parameter for tissue formation and function, different concentrations of CMs were tested ranging from 5 × 10^6^ - 25 × 10^6^ cells/mL each composed of ~ 90% hiPSC-CMs and ~ 10% human foreskin fibroblasts (hFFs) (Fig. 3a–d). Notably, the addition of cells to the fibrinogen and thrombin solution even at the highest cell concentration tested did not impair the polymerization process and was fully compatible with proper tube formation (Fig. 3e). Furthermore, the centrifugal force of approximately 100 × g employed in the process did not hamper the cell viability as indicated by persistent nuclear RedStar fluorescence and active calcium signaling. The initial cell concentration applied correlates with the observation of RedStar positive cells quantified from images of the outer wall of the respective tubes (Fig. 3f). For the highest cell number tested, representing about 22.5 × 10^6^ CMs/mL, a quantification of the cell density was not feasible as the multi-layered structure hindered an accurate discrimination of single cells. The highest cell concentration tested was notably required to retrieve tubes showing functional CMs coupling, as revealed by the synchronized oscillation of the GCamP6f signal 7 days after fabrication by RMT (Fig. 3g, h, supplemental movie1).

### Rotating mold technology (RMT) promotes functional CM coupling

**Figure 4:**
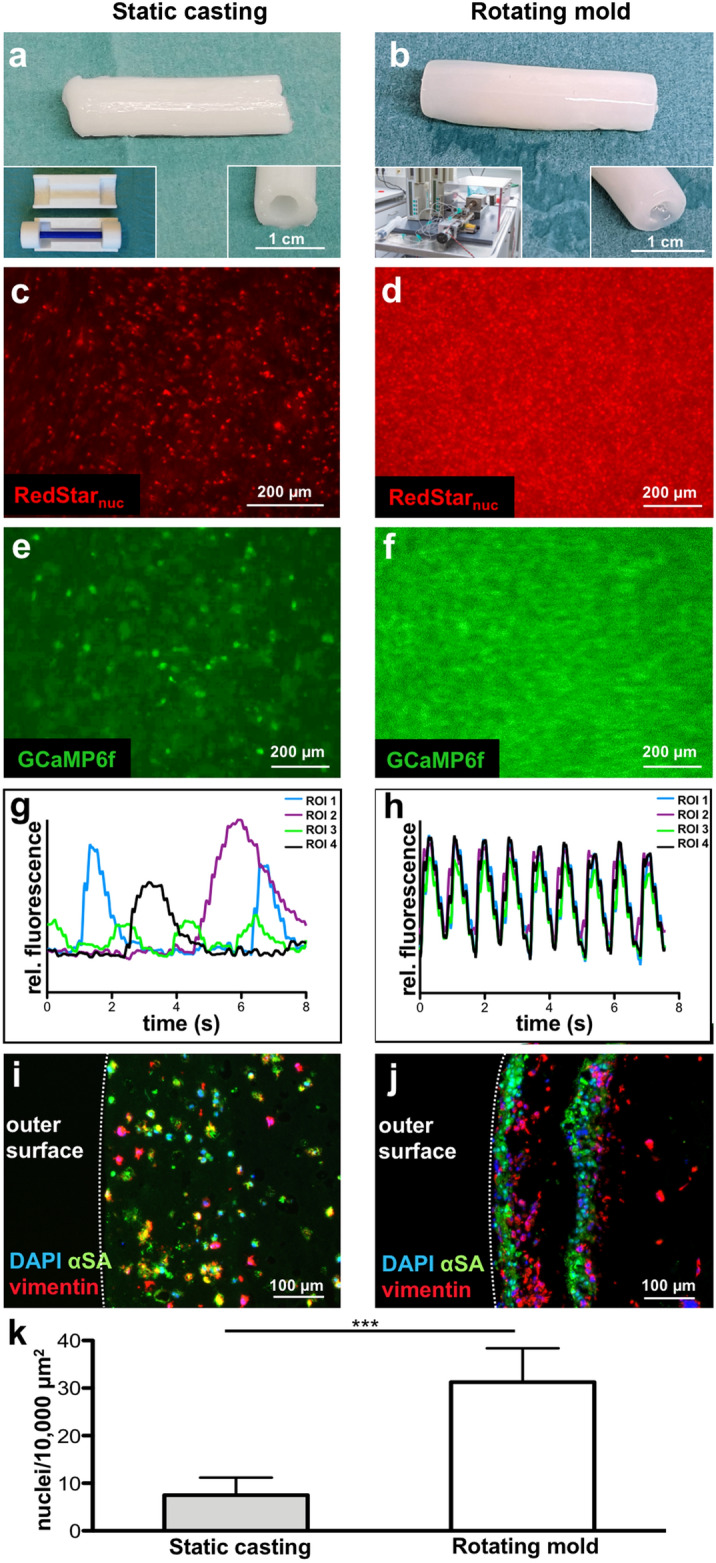
Rotation mold technology (RMT) facilitates CM coupling in contrast to static casting (SC) resulting in isolated CMs. (**a**,**c**,**e**,**g**,**i**) SC-tube. (**b**,**d**,**f**,**h**,**j**) RMT-tube. Tubes were fabricated employing the same cell concentration. (**a**) Macroscopic appearance of SC-tube. Left inset: 3D printed mold for static casting. Right inset: Lumen of SC-tube. (**b**) Macroscopic appearance of RMT-tube. Left inset: Machine for fabrication (copyright: Karin Kaiser/MHH). Right inset: Lumen of RMT-tube. (**c, d**) View onto the outer tube surface revealing nuclear RedStar fluorescence after 8 days of cultivation in SFM. (**e, f**) GCaMP6f peak fluorescence of Ruby CMs after 8 days of cultivation in SFM. Still images from movie. (**g, h**) Calcium fluctuations represented by GFP signal intensity were analyzed over time with ImageJ employing the recording shown in supplemental movie 2 and 3, respectively. Four regions of interest (ROI) were randomly selected. (**i, j**) Immunofluorescence of cross-sections stained for sarcomeric α-actinin (αSA, green), vimentin (red), and DAPI (blue). The dashed line represents the outer surface of the fibrin tube. (**k**) Quantification of nuclei per area for cross-sections from SC-tubes and RMT-tubes, respectively. Four regions of interest (ROI) were selected. For SC-tubes ROI were selected randomly, for RMT-tubes areas in the cell dense layers were selected. Mean and SD is depicted. *** = p < 0.001. Scale bar: (**a**,**b**) 1 cm for inset and main picture, (**c**–**f**) 200 µm, (**i**,**j**) 100 µm.

To evaluate the impact of RMT on cell distribution and functionality of cardiac tubes, our technology was compared to a “static casting” (SC) approach. Therefore, 3D printed casting molds were designed for generating tubes with an outer diameter of 8 mm and a wall thickness of 2 mm (Fig. 4a), closely reflecting dimensional properties of tubes generated by RMT (Fig. 4b). Applying the highest concentration of 25 × 10^6^ cells/mL tested above for both technologies, revealed substantial differences regarding the cell distribution and functionality in the resulting tissues (Fig. 4). When observing the outer surface of SC-tubes, CMs were rather loosely distributed accompanied by uncoordinated flashing activity of the calcium sensor in the isolated, individual CMs (Fig. 4c, e, g, supplemental movie 2). This distribution was confirmed over the entire wall of the fibrin tube in cross-sections (Fig. 4i). In contrast, when analyzing the outer surface of RMT-tubes after an equivalent time of tissue formation (i.e. 8 days after fabrication) a highly compact CM distribution was observed similar to the previous experiment (Fig. 3) showing a synchronized activity of GCamPf6 (Fig. 4d, f, h, supplemental movie 3). However, analysis of cross-sections revealed a sandwich-like structure of individual cell-dense layers, separated by essentially cell-free matrix (Fig. 4j). Quantifying the nuclei per area in these cross-sections from SC-tubes and RMT-tubes, here restricted to the cell-dense layer, confirmed the increase in cell density by the RMT process (Fig. 4k). The layered tissue characteristics in RMT-tubes may reflect the RMT dependent process properties, as RMT is based on moving the applicator dispensing the liquid cell-fibrinogen and cell-thrombin mixture, respectively, along the axis of the tube in repeated cycles. Nevertheless, our histological and functional analysis strongly indicates that RMT-induced formation of compact cell layers promotes the formation of physiologically coupled cardiac tissues.

### Compaction of fibrin tubes depends on hFF content and medium composition

**Figure 5:**
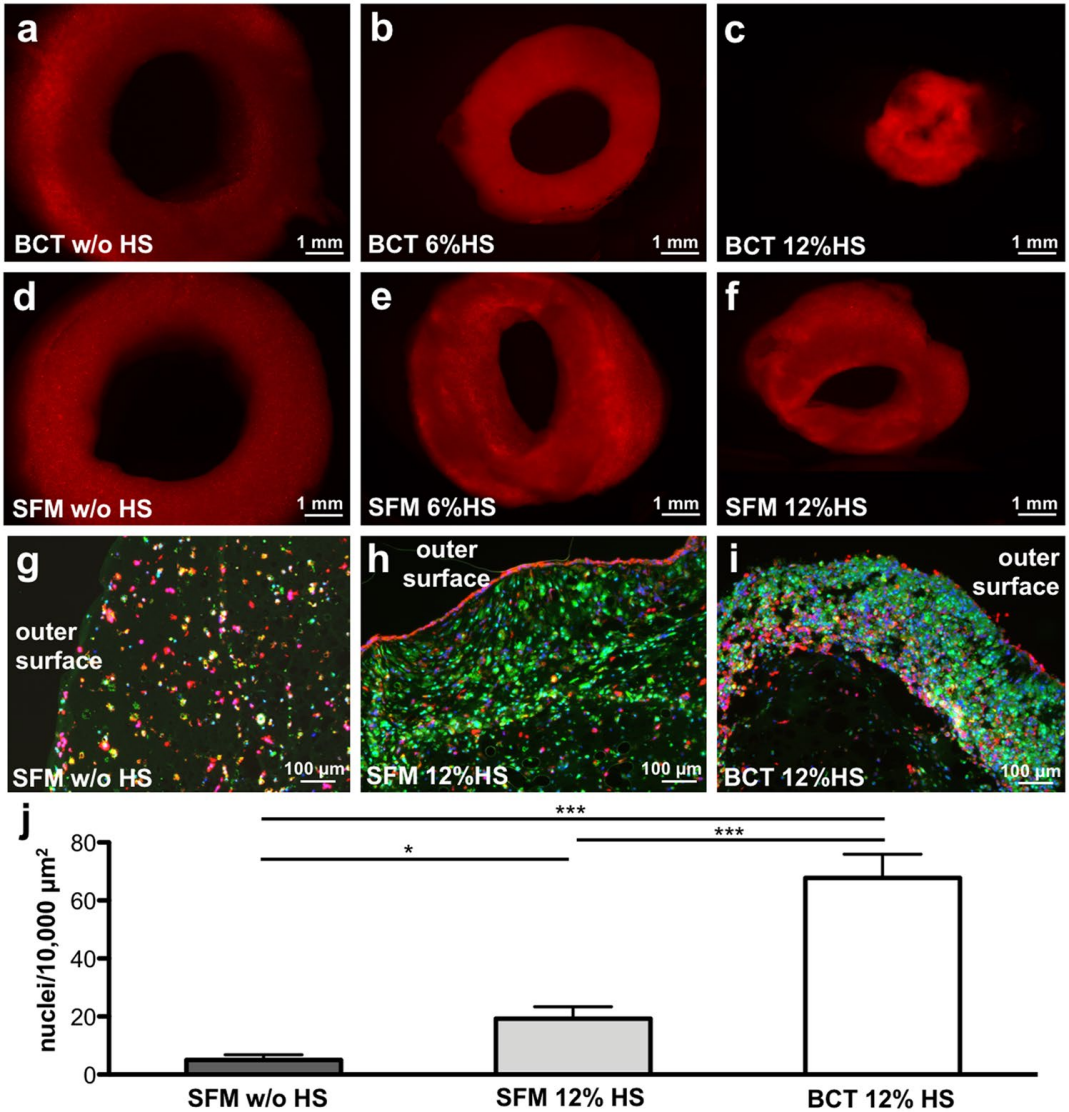
Compaction of SC-tubes is medium and horse serum content dependent. SC-tubes were cut in rings and subjected to different medium compositions for 7 days. (**a–c**) BCT medium. (**d–f**) SF medium. (**a**,**d**) No horse serum (HS). (**b**,**e**) 6% HS. (**c**,**f**) 12% HS. (**g–i**) Immunofluorescence of cross-sections of rings in (**d**), (**f**), (**c**), respectively, stained for sarcomeric α-actinin (αSA, green), vimentin (red), and DAPI (blue). (**j**) Quantification of nuclei per area for cross-sections from of rings in (**d**), (**f**), (**c**), respectively. Four regions of interest (ROI) were selected in the cell containing regions. Mean and SD is depicted. * = p <0.05, *** = p < 0.001.Scale bar: (**a**–**f**) 1 mm, (**g**–**i**) 100 µm.

In previous research employing collagen and Matrigel™ as a matrix for the generation of sheet-like bioartificial cardiac tissues (BCTs), the constructs were cultivated in 12% horse serum (HS) containing “BCT medium” (BCTM)^17^. Employing such medium for fibrin tube cultivation resulted in a strong alteration/compaction of the 3D structure within 8 days, although the fibrinolytic inhibitor aprotinin (200 KIU/mL) was added. Such compaction, mainly as a result of fibrinolysis caused by hFFs, resulted in progressive tube disintegration while the alternative cultivation in a serum-free media formulation (SFM)^28^ did not induce this process (data not shown). To systematically investigate the impact of media composition, SC-tubes containing 22.5 × 10^6^ Ruby-CMs/mL and 2.5 × 10^6^ hFFs/mL were cut into rings of equal length and subjected to cultivation in BCTM and SFM with increasing amounts of HS (Fig. 5). Compaction-associated effects were observed for up to 7 days, revealing increased tissue shrinkage in reply to higher HS content in both media (Fig. 5a–c, d–f). Without HS addition, no shrinkage was observed irrespective of the basal medium (Fig. 5a, d). However, when combined with HS, the compaction was more pronounced in BCTM compared to SFM. Immunofluorescence staining of cross-sections revealed an increase in cell density in response to the compaction process (Fig. 5g–i). Quantification of nuclei per area in the respective cross-sections confirmed the increase of nuclei density upon compaction (Fig. 5j). This tissue compaction effect was notably accompanied by the synchronization of the Ca-sensor activity (data not shown), corroborating the importance of proximal cell–cell contacts for the electrophysiological coupling of CMs in cardiac tissues.

**Figure 6:**
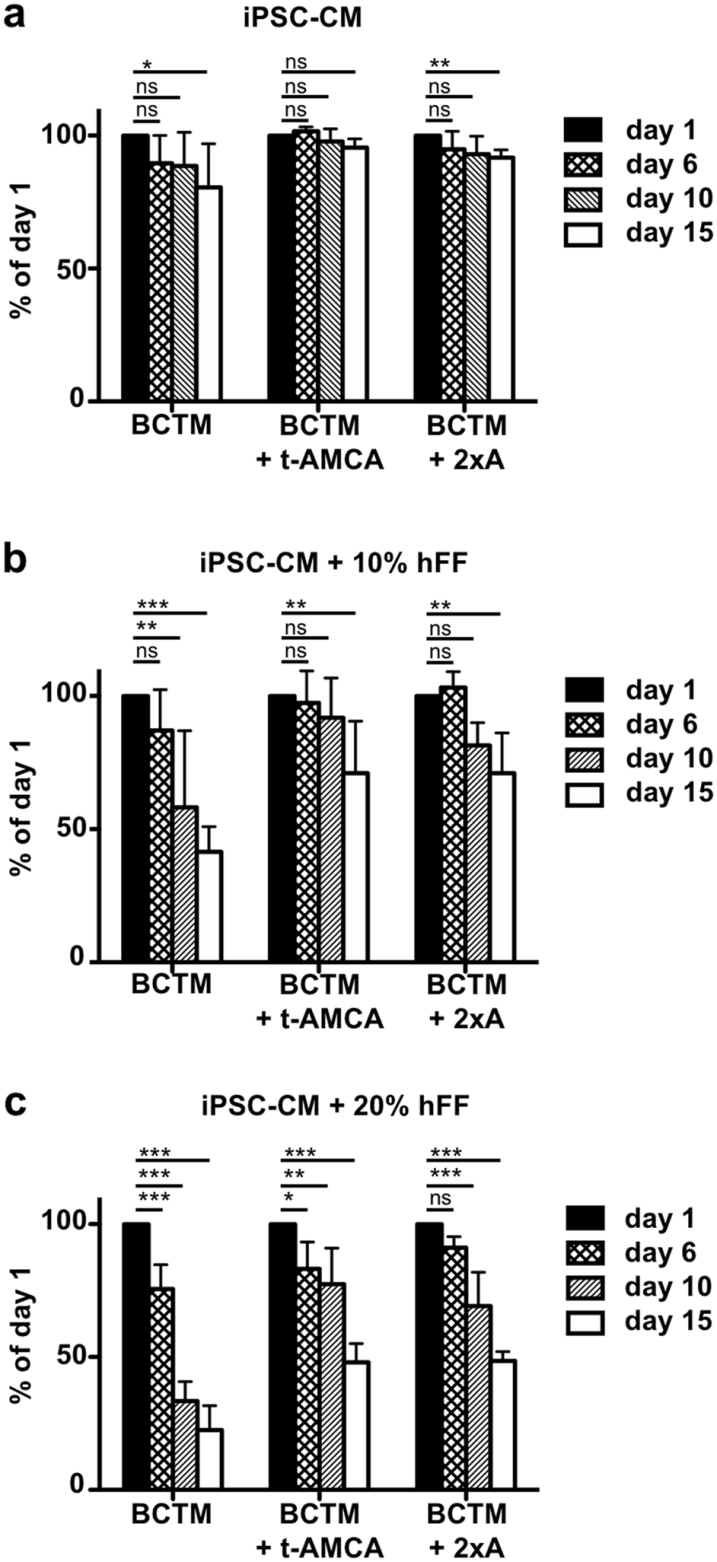
Compaction of SC-tubes is influenced by hFF content and inhibitor addition. SC-tubes containing different amounts of hFFs, (**a**) no hFFss, (**b**) 10% hFFs, (**c**) 20% hFFs, were cut into rings and subjected to different inhibitors for 15 days (BCTM: 200 KIU/mL aprotinin; BCTM + t-AMCA: 200 KIU/mL aprotinin, 200 µM tranexamic acid (t-AMCA); BCTM + 2xA: 400 KIU/mL aprotinin). Brightfield pictures were taken and the cross-sectional area of the rings was measured in AxioVision SE64 Rel. 4.9 and expressed as percentage of the day 1 value. N = 5, Mean and SD is depicted. ns = p > 0.05, * = p < 0.05, ** = p < 0.01, *** = p < 0.001.

The presence of fibroblasts is also known to play an important role in functional cardiac tissue formation *in vitro*^17^. Thus, the contribution of hFFs in the compaction process as well as fibrinolytic inhibition by t-AMCA or aprotinin supplementation was investigated by employing rings cut from SC-tubes (Fig. 6). The base medium contained 6% HS and 200 KIU/mL aprotinin. When entirely omitting hFFs from the cell–matrix composition, only minor compaction occurred, indicated by the reduction of cross-sectional area over time, independent of the inhibitors used (Fig. 6a), while hFF addition resulted in prominent, dose-dependent shrinkage (Fig. 6b, c). The addition of t-AMCA or doubling the concentration of aprotinin led to a comparable and slower degradation of the ring structures, resulting in a twofold larger cross-sectional area compared to BCTM controls on day 15. As a result of this systematic time-dependent investigation, it was decided to utilize a content of 10% hFFs for tube formation, followed by cultivation in BCTM containing t-AMCA in further experiments.

### Conceptual re-design and scale-up results in a highly versatile device prototype for the controlled production of larger fibrin tubes

**Figure 7:**
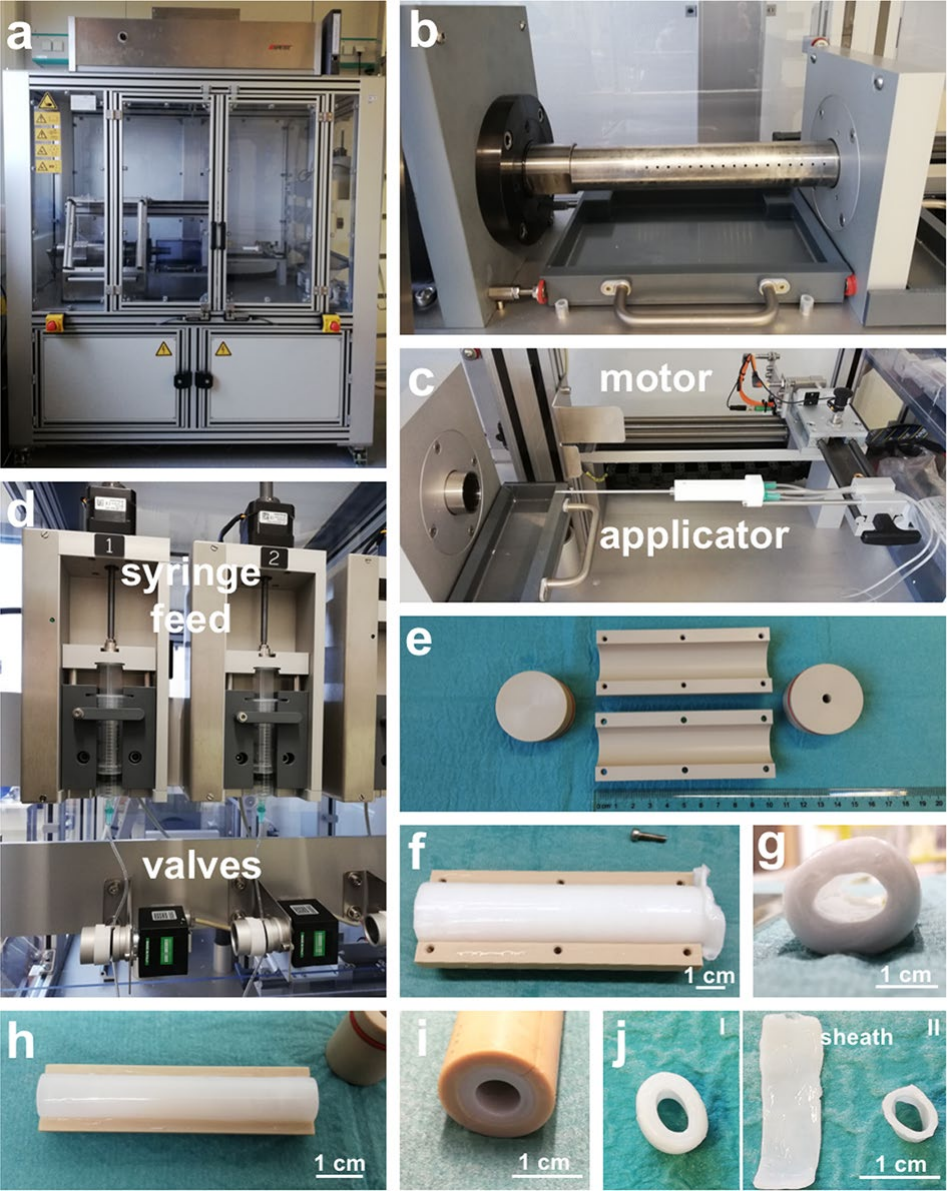
Automated machine for the production of fibrin tubes. (**a**) View of the machine. (**b**) Stainless steel pipe fitted on the motor for rotation. (**c**) Applicator equipped with 2 cannulas connected to drip lines. The motor for moving the applicator is located in the back. (**d**) Automated syringe feed with valves for closing and opening the lines. (**e**) PEEK mold for tube production. (**f**) Fibrin tube with a length of 10 cm and a diameter of 22 mm produced with the machine. Top view with one half shell removed. (**g**) Side view into the lumen of the tube in (**f**). (**h**) Fibrin tube produced in a mold with a length of 6 cm and an outer diameter of 11 mm. Top view with one half shell removed. (**i**) View into the lumen of a tube in the mold. (**j**) (I) Ring cut off from a tube produced with an outer sheath. (II) Same Ring as in (I) after the removal of the sheath which was cut open and placed on the left side of the ring. Scale bar: (**f**–**j**) 1 cm.

To overcome both technical limitations regarding the control of numerous process parameters related to RMT and limitations regarding the production of tubes with large diameters, a new machine was required. The new device prototype including the respective control software was designed and built together with the MHH research workshop (Fig. 7a–d). The set-up comprised the use of polyetheretherketone (PEEK)-based molds of variable dimensions, allowing the rotation mold production of tubes with 10 cm in length and a diameter of up to 22 mm, which was readily achieved in proof-of-concept experiments (Fig. 7e–g). However, by adjustment of mold dimensions, different lengths and diameters of tubes can be realized (Fig. 7h–j), whereby 6 cm in length and 11 mm in outer diameter were chosen for further experiments employing iPSC-CMs and hFFs. In addition, slight changes in the production process were implemented which were enabled by the new device. As noticed in previous experiments, cell loss was unavoidable due to centrifugal forces pushing cell-containing liquid out of the mold until sufficient polymerization of fibrin acted as a sealant. For an improved fabrication, a cell-free sheath was produced in the first step, directly followed by the application of iPSC-CMs and hFFs containing fibrinogen and thrombin solution forming a tube-in-tube structure with the possibility to remove the outer sheath (Fig. 7j).

### Tube Production with the new device combined with the sheath strategy leads to functional cardiac tubes of substantial dimensions

**Figure 8:**
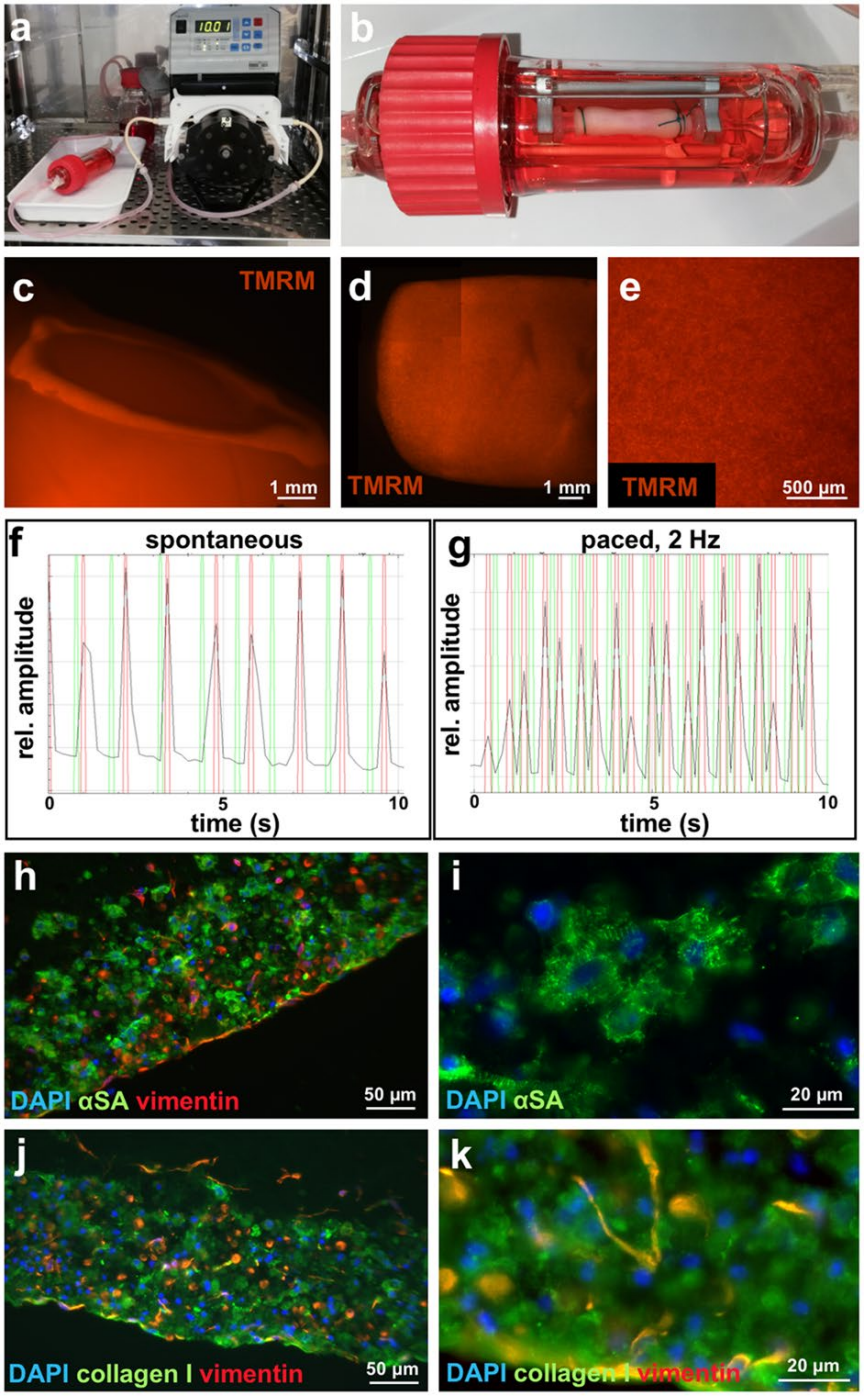
Analysis of fibrin tubes fabricated with the new device and cultivated under pulsatile flow. (**a**) Bioreactor set-up for pulsatile flow. (**b**) Fibrin tube mounted into a bioreactor. (**c**) View into the lumen of the tube stained with TMRM. (**d**) Side view of a tube stained with TMRM. Four images were stitched together. (**e**) Picture with high magnification of the tube in (**d**). (**f**,**g**) Videos from contracting fibrin tubes stained with TMRM were recorded, imported into ImageJ and analyzed with the MYOCYTER macro to reveal the beating frequency. The amplitude is depicted in black, the maxima in red and the minima in green. (**f**) Analysis of spontaneous beating fibrin tube. (**g**) Analysis of tube from (**f**) paced at 2 Hz. (**h–k**) Immunofluorescence of cross-sections. (**h**,**i**) Staining for sarcomeric α-actinin (αSA, green), vimentin (red), and DAPI (blue). (**j**,**k**) Staining for collagen I (green), vimentin (red), and DAPI (blue). Scale bar: (**c**,**d**) 1 mm, (**e**) 500 µm, (**h**,**j**) 50 µm, (**i**,**k**) 20 µm.

To ensure the general applicability of our method, an additional iPSC line MHHi001-A^29^ was used. In addition, an analysis of bioartificial cardiac tissue (BCT) generated by a different technique^30^ revealed that force generation of these tissues depends on the iPSC cell lines used for CMs production. Tissues derived from CMs originating from the initially employed iPSC cell line MHHi001-A-5 exhibited lower forces compared to BCTs derived from the parental iPSC line MHHi001-A (unpublished data). As the iPSC line MHHi001-A does not carry any reporter, cell viability was demonstrated by TMRM staining and further applied throughout the entire cultivation time for imaging of vital cells. RMT-tubes (6 cm in length, 11 mm outer diameter) produced with an outer sheath were equipped with connectors and subjected to pulsatile flow of 10 mL/min in a bioreactor at the frequency of 1 Hz (Fig. 8a, b, supplemental movie 4). Employing 10% of hFFs with a total number of 150 × 10^6^ cells per tube and cultivation in BCTM with t-AMCA enabled prolonged cultivation of up to 5 weeks without substantial shrinkage of the fibrin tube. TMRM staining revealed the even distribution of cells in circumferential and longitudinal direction (Fig. 8c-e). After discontinuing the pulsatile flow, spontaneous contractions of the fibrin tubes were visible. Contractions were recorded at different positions and analyzed revealing 65 ± 9 beats per minute (Fig. 8f). Further functionality of the iPSC-CMs was demonstrated by their proper reaction to electrical stimulation via pacing at a frequency of 2 Hz (Fig. 8g). Staining of cross-sections revealed an outer cell-dense layer of CMs and fibroblasts (Fig. 8h) with CMs exhibiting cross-striations (Fig. 8i). Deposition of collagen I was demonstrated revealing intense staining for collagen when co-localized with vimentin expressing fibroblasts (Fig. 8j, k) while showing faint staining in regions negative for vimentin. A cell density of 51 ± 7.5 nuclei/10,000 µm^2^ was determined by automatic counting of cross-sections; this value is slightly higher compared to the cell-dense layers in the RMT-tubes produced in the previous set-up (Fig. 4k).

## Discussion

Here, we have demonstrated the feasibility to produce large, tubular fibrin constructs based on commercially available human fibrinogen and thrombin employing iPSC-CMs using RMT. Although the use of fibrinogen from FFP seems reasonable, inhomogeneous fibrin tubes were produced from cryoprecipitated fibrinogen. The isolation by cryoprecipitaion results presumably in fibrinogen with a high content of impurities, which might alter the rheological behavior leading to an uneven distribution along the axis of the mold and therefore resulting in the observed heterogeneity in Young’s modulus along individual tubes (Fig. 2). Hence, commercially available fibrinogen (cFb) significantly increased the experimental reproducibility resulting in rather homogenous tube formation by RMT, cFb was employed in further experiments. Through the centrifugal forces in the fabrication process CMs are concentrated and brought into close proximity to each other facilitating the coupling of CMs as early as day 3 of cultivation as indicated by the activity of the calcium sensor GCaMP6f (Fig. 3, supplemental movie 1). Comparison of RMT-tubes to SC-tubes employing the same cell concentration of 25 × 10^6^ cells/mL clearly demonstrated the increase of cell density in the rotation process (Fig. 4). The final cell density is an important aspect for functional tissue formation as local cell–cell communication via a variety of secreted factors as well as direct CM-CM communication via gap junctions is crucial for the maturation of CMs and impulse conduction^31^. In the final set-up the applied cell concentration was even increased to 50 × 10^6^ cells/mL reaching the technical limit of the set-up as higher cell densities impaired the transport of solutions via the drip line (data not shown).

Further analysis of the impact of the ratio of hFFs as well as medium components on remodeling of the fibrin matrix revealed that a combination of 10% hFFs and the addition of t-AMCA results in moderate compaction enabling the culture of fibrin tubes for up to 5 weeks without losing their initial structure. Aiming at the future implementation of iPSC-derived cardiac fibroblasts, this data would need to be revised as Boucard et al. reported the dependence of fibrin degradation on fibroblast origin^32^. They observed that proteases such as MMP1, MMP3, and urokinase, which are directly or indirectly involved in fibrinolysis, were expressed at varying levels depending on the derivation of fibroblasts^32^. In the same study, it was demonstrated that aprotinin exhibited a dose-dependent effect on degradation, which was reduced by a factor of 2 when increasing the aprotinin concentration from 20 µg/mL to 40 µg/ml^32^. A similar trend was observed in our study when increasing the aprotinin content from 200 KIU/ml to 400 KIU/mL in the ring-based assay (Fig. 6).

Prevention of fibrin degradation is not only important in vitro but of utmost importance in vivo as native fibrin will be degraded as soon as it is implanted into the body. Engineering a fibrin-based construct suitable for transplantation requires either the modification of fibrin per se, e.g. via crosslinking, or the replacement of fibrin by extracellular matrix proteins deposited by cells during the in vitro cultivation. Indeed, Syedain et al. generated implantable tissue-engineered blood vessels as well as heart valves by prolonged cultivation of fibroblasts replacing the initial fibrin matrix by collagen^33,34^. In our study, collagen deposition by hFFs and also by CMs was demonstrated (Fig. 8) but further analysis would be required to determine if the retrieved tissue exhibits sufficient stability. Collagen deposition could be increased by activation of fibroblasts or by increasing their amount with the drawback of increased fibrinolytic activity as demonstrated in our study (Fig. 6). These findings also highlight the importance of thoroughly fine tuning the fibrinolytic and anti-fibrinolytic components in fibrin-based cardiac tissue engineering.

With future clinical applications in mind, we have employed a rather high fibrinogen concentration of 30 mg/mL aiming at establishing a mechanically relatively strong matrix. Nanoindentation revealed a Young’s modulus of 2.6 ± 0.5 kPa on average, which is well in line with a value of 1.0 ± 0.3 kPa for fibrin based hydrogels with a concentration of 15 mg/mL fibrinogen reported by Jung et al. employing a similar nanoindenter^35^. Jung et al. observed a large scattering of the values retrieved by nanoindentation, which we detected in our data sets as well. The high deviation of the values seems to be inherent to the technology i.e. measuring fibrous samples with probes in the micrometer range. Although the Young’s modulus still resembles values for rather soft tissues, tubes could be secured with a surgical thread onto plastic connectors for implementation in a perfusion bioreactor and could withstand subsequent stimulation by pulsatile flow (Fig. 8).

In a recent publication Koehne et al. followed a casting strategy for the generation of tubular heart tissue employing bovine fibrinogen with a final concentration of 10 mg/mL. Koehne et al. employed a silicon tubing as well as velcro rings to secure tube integrity^12^. In addition, the necessity for a silicon support tube hindered the assessment of burst pressure in their setting. Also others used a tubular support such as an octagonal column^9^ or a decellularized human umbilical artery^10^ for cardiac tubular tissue engineering. In these approaches CMs were introduced as cell sheets generated by culture on thermoresponsive tissue culture plates^9^ or cultured on decellularized slices of porcine ventricular heart tissue^10^ wrapped around the tubular support. Williams et al. used thermoresponsive nanofabricated substrates with cell-sheet stacks wrapped into a cylindric shape which is stabilized by casting and crosslinking a hydrogel around the sheets^11^. All these studies have in common that a technique was implemented to produce a cell dense layer to foster the coupling and functionality of employed CMs. In the current study, this is easily achieved by applying a high density of up to 50 × 10^6^ cells/mL within the rotation process without labour intensive pre-cultivation of cell sheets. Remarkably, the initial cell density used in our study is much higher compared to reports by Koehne et al.^12^ (18 × 10^6^ cells/mL) and Querdel et al.^36^ (15 × 10^6^ cells/mL) both following a casting approach. Through the application of centrifugal forces inherent in our approach, the cells are in addition even more concentrated at the outer perimeter of the tube probably approaching the cell density of the native human heart with 100 × 10^6^ cells/mL^37^. Functionality of our fibrin-based cardiac tubes was demonstrated via their ability to spontaneously contract with 65 ± 9 beats per minute correlating well with the mean real-world heart rate of 79 ± 15 beats per minute recently determined in the eHeart study^38^. Furthermore, the tissues were able to react to electrical stimuli with increasing their beating frequency according to the pacing (Fig. 8).

The reported survival and functionality of iPSC derived CMs has been demonstrated for up to 100 days for hiPSC derived 3D cardiac aggregates^39^ and organoids^40^ in vitro. In vivo, the presence of transplanted hiPSC-CM has been revealed for at least 3 months in large animal models shown by us^41^ and others^42^, providing meaningful hints for the long-term engraftment and phenotypic stability of iPSC-CMs and thus supporting the envisioned therapeutic concept. So far, the described approaches have resulted in rather small constructs with 1.8 cm in length and 6 mm inner diameter published by Koehne et al. being the largest one reported^12^. The current dimensions of our approach may not yet fulfill the clinical requirements for a biological cardiac assist device, but our constructs are generally suitable for first planned functionality tests in ex vivo perfused porcine hearts. As our approach is straightforward regarding the further scalability to larger formats (Fig. 7), dimensions suitable for preclinical testing in animal models can be achieved. Clearly, with the increase in size, nourishment of cell-containing constructs represents a major challenge that needs to be addressed. However, in combination with the advanced upscalable bioreactor technology for the GMP-compliant production of large amounts of iPSC derivatives^26,43^ the presented technology paves the way for the production of functional cardiac tubes for clinical translation envisioned as biological cardiac assist device.

## Material and Methods

### Fibrinogen isolation

Fresh frozen plasma concentrates (FFP) unsuitable for clinical use were obtained from the blood bank at Hannover Medical School. Frozen plasma was thawed at room temperature and centrifuged at 800 × g for 12 min. The supernatant was discarded and the hydrous protein pellet was solubilized at 37 °C.

### Fibrin tube production

Initially, fibrin tubes were fabricated in a machine established by Aper et al.^23^ with a few modifications. A schematic of the process is depicted in Fig. 1. In brief, a rotating mold was driven by an electric motor. The apparatus consisted of an outer brass tube with 10 pairs of drill holes (diameter 0.3 mm, distance 1 cm) allowing the drainage of excess fluid, and two removable polyetheretherketone (PEEK) half shells inside. The cylindric mold was closed on both sides with PEEK stoppers with the front stopper provided with a hole enabling the insertion of an applicator. The applicator was driven by an electric motor enabling the movement in the mold along the axis. Two cannulas connected via drip lines to syringes driven by injectomats were inserted into the applicator. Thrombin and fibrinogen solutions were applied through these cannulas at a defined flow rate into the mold while the applicator was moved along the axis. As this apparatus allowed only a limited diameter for the tube and had limited control over movement speed and other parameters, a more sophisticated machine was built enabling the fabrication of fibrin tubes up to 34 mm in diameter (Fig. 6). Fully automated control over application speed off the solutions, movement of the applicator, and rotation speed was implemented allowing the execution of complex fabrication protocols.

For the fabrication of fibrin tubes two solutions were prepared independently. Commercially available human fibrinogen (Merck) and commercially available human thrombin (Merck) were solubilized in 0.9% NaCl. CaCl_2_ solution was added to the thrombin solution. Application of equal volumes of the two solutions resulted in final concentrations of 30 mg/mL fibrinogen, 15 U/mL thrombin, and 40 mM CaCl_2_. Process parameters for the old machine were the following: centrifugal force of 72–162 × g (strong variation due to the properties of the motor), application speed for both solutions of 1.25 mL/min, and applicator speed of 1 mm/s. A volume of 3 mL for each solution was applied. Process parameters for the new device were the following: centrifugal force of 125 × g, application speed for both solutions of 1 mL/min, and applicator speed of 1.33 mm/s. A total volume of 4.5 mL was applied for the fabrication of a cell-free sheath and a total volume of 3.5 mL was used for cell application.

### Nanoindentation

Pieces from the fabricated fibrin tubes were glued to a glass bottom dish, incubated for 30 min in PBS, and subjected to nanoindentation with PIUMA nanoindenter (Optics11, Amsterdam, The Netherlands) employing a 0.29 N/m cantilever-based probe with a tip radius of 49 µm. Measurements were done at room temperature. Per sample, 100 indentations were performed in a grid of 1 × 1 mm with a distance of 100 µm between individual indentations. The displacement profile is set to displace 20 µm from the automated near-surface definition. The data in the loading section of the load–displacement curve was used to determine the Young’s Modulus using a fit of all data points from the contact point to 7 µm indentation depth respecting the Hertz model assumption of a parabolic indenter (max. depth for calculation equals to 16% of tip radius). Two to three pieces per tube were analyzed.

### Cardiac differentiation

Single hiPSC were seeded in E8 medium supplemented with 10 µM Y27632 and Pluronic F-68 at 0.1% in single-use 500 ml Spinner Flasks (Corning) placed on a Cimarec spinner platform (Thermo Fisher, 60 rpm). Subsequently, 48 h after inoculation the medium was replaced with CDM3 medium (Burridge et al., 2014; RPMI1640; 2 mM glutamine; 495 µg/ml human recombinant albumin (ScienCell, SC-OsrHSA); 213 µg/ml ascorbic acid (Sigma-Aldrich)) supplemented with 5 µM CHIR. After 24 h, medium was replaced by CDM3 supplemented with 5 µM IWP2. After 48 h, medium was replaced with CDM3 and was exchanged every 2–3 days. Cardiac differentiation > 90% was confirmed by FACS analysis for cardiac troponin T, myosin heavy chain, and sarcomeric α-actinin as previously described^26,27^. Cells at days 20–25 of differentiation were dissociated with STEMCELL technologies cardiomyocyte dissociation kit for 3–10 min at 37 °C as previously described^27^. Cells were automatically counted (Vi-Cell XR, Beckman Coulter) and distributed equally in fibrinogen and thrombin solution according to the number of viable cells.

### Fibroblast cultivation

Human foreskin fibroblast (hFF, SCRC-104, ATCC) were cultivated in DMEM (21969-035, Gibco) containing 2 mM L-glutamine, 10% FBS (10270106, Gibco) and MEM solution of non-essential amino acids (11140050, Gibco). Cells were released from plates by trypsin, automatically counted (Vi-Cell XR, Beckman Coulter), and equally distributed between fibrinogen and thrombin solution according to the number of viable cells.

### Cultivation of fibrin tubes

Fibrin tubes were either cultivated in bioartificial cardiac tissue medium (BCTM) composed of high glucose DMEM (Gibco#21969), horse serum (HS, Gibco#16050122, content according to the experimental setting), 2 mM L-glutamine, 100 U/mL penicillin, 100 µg/mL streptomycin, 10 µg/mL human insulin, 60 µM ascorbic acid (Merck), and 200 KIU/mL Aprotinin (Loxo) or in serum-free medium (SFM) composed of M199 as basal medium supplemented with 10 ng/mL VEGF-A, 10 ng/mL FGF-2, 0.1% ITS, 2 mM L-glutamine, 1% BSA, 50 µg/mL ascorbic acid, 0.2 µg/mL hydrocortisone, 0.1 mg/mL gentamicin, 100 U/mL penicillin, 100 µg/mL streptomycin, 200 KIU/mL aprotinin (Loxo), and horse serum (HS, content according to the experimental setting). For the first 24 h of cultivation the medium was supplemented with 5 µM CHIR. Medium was exchanged ever 2–3 days. In the bioreactor setting, medium was changed every 5–7 days.

### Tetramethylrhodamine, methyl ester (TMRM) staining

TMRM (T668, ThermoFisher Scientific) was added at a concentration of 50 nM to the cultivation medium for visualization of living cells in the constructs.

### Histology

Immunofluorescence staining was performed according to standard protocols and is described below. Fibrin rings or tubes were fixed with 4% paraformaldehyde in PBS at room temperature for 20 min followed by 3 washing steps using PBS. For cryosection preparation specimens were transferred into optimal cutting temperature compound (OCT) and left for 24 h at 4 °C. The OCT-embedded constructs were frozen in liquid nitrogen and stored at − 80 °C. The frozen constructs were mounted on a cryostat and 5 µm cryosections were prepared and transferred onto superfrost glass slides. The cryosections were dried at RT for 2 h before storage at -80 °C. Specimens were permeabilized with 0.25% Triton X-100 for 1 h at room temperature. Blocking was performed with 2% donkey serum in PBS for 20 min at room temperature followed by overnight incubation with primary antibodies: anti-vimentin antibody (ab92547, abcam, 1:500), anti-α-actinin (sarcomeric) antibody (A7811, Sigma-Aldrich, 1:800), and anti-collagen type I (C2456, Sigma-Aldrich, 1:500) at 4 °C. On the next day, sections were washed three times with PBS and incubated with Cy3 conjugated donkey anti-goat antibody (705-165-147, Jackson ImmunoResearch, 1:300), and Alexa Fluor 488 conjugated donkey anti-mouse antibody (715-545-151, Jackson ImmunoResearch, 1:300) for 30 min at room temperature. Samples were washed again three times with PBS. Nuclei were counterstained with 4′,6-diamidino-2-phenylindole (DAPI) (1 μg/mL in PBS) for 15 min at room temperature. After additional washing steps with PBS, sections were covered with fluorescent mounting medium and glass coverslips. Stained samples were analyzed with the Axio Observer A1 microscope and the Zeiss Observer Z1 microscope. For better visualization of the sarcomeric α-actinin signal the unsharp tool in AxioVision SE64 Rel. 4.9 was used with preset standard values.

### Image analysis for cell density

Images taken from TMRM stained or RedStar expressing living tissues and sections stained for DAPI were analysed with ImageJ. Images were transformed in 8bit images and the local threshold (Bernsen) was adjusted. Resulting images were converted into binary images and objects were separated with the watershed function. Four regions of interest (ROI) with the same area were randomly chosen and the particle content was counted.

### Measurement of cross sectional area

Living tissues were observed using the Stereo Microscope Discovery V8. (Zeiss). Brightfield pictures of rings cut form static cast tubes were taken and the cross sectional area of the rings was measured in AxioVision SE64 Rel. 4.9 and expressed as percentage of the day 1 value. Three independent experiments were analyzed.

### Analysis of GFP signal intensity over time

Videos of the GFP signal were captured at 60 frames per second using the Stereo Microscope Discovery V8. (Zeiss). Videos were imported into ImageJ. The Plugin “Time series analyzer” was employed to retrieve the average intensity value for 4 randomly chosen regions of interest (ROI) over time. GraphPad Prism was used to transform the data into relative fluorescence values and plot the retrieved values over time.

### Pacing

Platinum wires connected to a custom-made pacing device developed in collaboration with the MHH Research Workshop were submerged in the culture medium. Biphasic pulses of 25 V were elicited at a frequency of 2 Hz and the presence of current was verified by a custom-made software (MHH Research Workshop). Videos of TMRM stained fibrin tubes (spontaneously contracting and paced) were taken at 5 frames per second with AxioObserver Z1 (Zeiss). Retrieved videos were imported into ImageJ and analyzed with MYOCYTER macro^44^.

### Documentation

Living tissues were observed using the Stereo Microscope Discovery V8. (Zeiss).

### Statistical analyses

Statistical analyses was performed with Prism. A one-way ANOVA test with Bonferroni’s Multiple Comparison post-test was used to analyze the differences between groups (Fig. 1e,d; Fig. 2e, Fig. 4j). An unpaired t-test was used for analyes of data depicted in Fig. 3K. For experiments depicted in Fig. 5 a repeated measures ANOVA with Dunnett’s Multiple Comparison post-test was used to analyze the differences compared to day 1. A p-value < 0.05 was considered statistically significant. Mean values with SD are reported.

### Supplementary Information

Supplementary Information 1.

Supplementary Video 1.

Supplementary Video 2.

Supplementary Video 3.

Supplementary Video 4.

## Data Availability

The datasets generated during and/or analyzed during the current study are available from the corresponding author on reasonable request.

## References

[1] Shadrin IY (2017). Cardiopatch platform enables maturation and scale-up of human pluripotent stem cell-derived engineered heart tissues. Nat. Commun..

[2] Tiburcy M (2017). Defined engineered human myocardium with advanced maturation for applications in heart failure modeling and repair. Circulation.

[3] Jackman CP, Carlson AL, Bursac N (2016). Dynamic culture yields engineered myocardium with near-adult functional output. Biomaterials.

[4] Miyagawa S (2022). Case report: Transplantation of human induced pluripotent stem cell-derived cardiomyocyte patches for ischemic cardiomyopathy. Front. Cardiovasc. Med..

[5] Kawamura T (2023). Safety confirmation of induced pluripotent stem cell-derived cardiomyocyte patch transplantation for ischemic cardiomyopathy: First three case reports. Front. Cardiovasc. Med..

[6] Sridharan D (2023). Preclinical large animal porcine models for cardiac regeneration and its clinical translation: Role of hiPSC-derived cardiomyocytes. Cells..

[7] Kishino Y (2023). Cardiac regenerative therapy using human pluripotent stem cells for heart failure: A state-of-the-art review. J. Card. Fail.

[8] MacQueen LA (2018). A tissue-engineered scale model of the heart ventricle. Nat. Biomed. Eng..

[9] Tsuruyama S, Matsuura K, Sakaguchi K, Shimizu T (2019). Pulsatile tubular cardiac tissues fabricated by wrapping human iPS cells-derived cardiomyocyte sheets. Regen. Ther..

[10] Park J (2020). Modular design of a tissue engineered pulsatile conduit using human induced pluripotent stem cell-derived cardiomyocytes. Acta Biomater..

[11] Williams NP (2020). Engineering anisotropic 3D tubular tissues with flexible thermoresponsive nanofabricated substrates. Biomaterials.

[12] Kohne M (2022). A potential future Fontan modification: Preliminary in vitro data of a pressure-generating tube from engineered heart tissue. Eur. J. Cardiothorac. Surg..

[13] Bliley J (2022). FRESH 3D bioprinting a contractile heart tube using human stem cell-derived cardiomyocytes. Biofabrication.

[14] Guyette JP (2016). Bioengineering human myocardium on native extracellular matrix. Circ. Res..

[15] Noor N (2019). 3D printing of personalized thick and perfusable cardiac patches and hearts. Adv. Sci. (Weinh).

[16] Kupfer ME (2020). In situ expansion, differentiation and electromechanical coupling of human cardiac muscle in a 3D bioprinted, chambered organoid. Circ. Res..

[17] Kensah G (2013). Murine and human pluripotent stem cell-derived cardiac bodies form contractile myocardial tissue in vitro. Eur. Heart J..

[18] Ke M (2024). Construction of millimeter-scale vascularized engineered myocardial tissue using a mixed gel. Regen. Biomater..

[19] Goldfracht I (2019). Engineered heart tissue models from hiPSC-derived cardiomyocytes and cardiac ECM for disease modeling and drug testing applications. Acta Biomater..

[20] Kaiser NJ, Kant RJ, Minor AJ, Coulombe KLK (2019). Optimizing blended collagen-fibrin hydrogels for cardiac tissue engineering with human iPSC-derived cardiomyocytes. ACS Biomater. Sci. Eng..

[21] Park CH, Woo KM (2018). Fibrin-based biomaterial applications in tissue engineering and regenerative medicine. Adv. Exp. Med. Biol..

[22] Mannucci PM (1998). Hemostatic drugs. N. Engl. J. Med..

[23] Aper T (2016). Novel method for the generation of tissue-engineered vascular grafts based on a highly compacted fibrin matrix. Acta Biomater..

[24] Regenberg MC, Wilhelmi M, Hilfiker A, Haverich A, Aper T (2023). Development, comparative structural analysis, and first in vivo evaluation of acellular implanted highly compacted fibrin tubes for arterial bypass grafting. J. Mech. Behav. Biomed. Mater..

[25] Haase A (2021). Establishment of MHHi001-A-5, a GCaMP6f and RedStar dual reporter human iPSC line for in vitro and in vivo characterization and in situ tracing of iPSC derivatives. Stem Cell Res..

[26] Halloin C (2019). Continuous WNT control enables advanced hPSC cardiac processing and prognostic surface marker identification in chemically defined suspension culture. Stem Cell Rep..

[27] Kriedemann NTW, Teske J, Mertens M, Franke A, Ullmann K, Manstein F, Drakhlis L, Haase A, Halloin C, Martin U, Zweigerdt R (2024). Standardized production of hPSC-derived cardiomyocyte aggregates in stirred spinner flasks. Nat. Protoc..

[28] Andree B (2019). Formation of three-dimensional tubular endothelial cell networks under defined serum-free cell culture conditions in human collagen hydrogels. Sci. Rep..

[29] Haase A, Gohring G, Martin U (2017). Generation of non-transgenic iPS cells from human cord blood CD34(+) cells under animal component-free conditions. Stem Cell Res..

[30] Kensah G (2011). A novel miniaturized multimodal bioreactor for continuous in situ assessment of bioartificial cardiac tissue during stimulation and maturation. Tissue Eng. C Methods.

[31] Tirziu D, Giordano FJ, Simons M (2010). Cell communications in the heart. Circulation.

[32] Boucard E (2022). The degradation of gelatin/alginate/fibrin hydrogels is cell type dependent and can be modulated by targeting fibrinolysis. Front. Bioeng. Biotechnol..

[33] Syedain ZH, Meier LA, Bjork JW, Lee A, Tranquillo RT (2011). Implantable arterial grafts from human fibroblasts and fibrin using a multi-graft pulsed flow-stretch bioreactor with noninvasive strength monitoring. Biomaterials.

[34] Syedain ZH (2021). Pediatric tri-tube valved conduits made from fibroblast-produced extracellular matrix evaluated over 52 weeks in growing lambs. Sci. Transl. Med..

[35] Jung SA (2023). Fibrin-dextran hydrogels with tunable porosity and mechanical properties. Biomacromolecules.

[36] Querdel E (2021). Human engineered heart tissue patches remuscularize the injured heart in a dose-dependent manner. Circulation.

[37] Chiu LLY, Radisic M (2013). Cardiac tissue engineering. Curr. Opin. Chem. Eng..

[38] Avram R (2019). Real-world heart rate norms in the Health eHeart study. NPJ. Digit. Med..

[39] Fleischer S, Jahnke HG, Fritsche E, Girard M, Robitzki AA (2019). Comprehensive human stem cell differentiation in a 2D and 3D mode to cardiomyocytes for long-term cultivation and multiparametric monitoring on a multimodal microelectrode array setup. Biosens. Bioelectron..

[40] Ergir E (2022). Generation and maturation of human iPSC-derived 3D organotypic cardiac microtissues in long-term culture. Sci. Rep..

[41] Gruh I (2024). Cell therapy with human iPSC-derived cardiomyocyte aggregates leads to efficient engraftment and functional recovery after myocardial infarction in non-human primates. BioRxiv..

[42] Liu YW (2018). Human embryonic stem cell-derived cardiomyocytes restore function in infarcted hearts of non-human primates. Nat. Biotechnol..

[43] Olmer R (2018). Differentiation of human pluripotent stem cells into functional endothelial cells in scalable suspension culture. Stem Cell Rep..

[44] Grune T, Ott C, Haseli S, Hohn A, Jung T (2019). The, "MYOCYTER"—Convert cellular and cardiac contractions into numbers with ImageJ. Sci. Rep..

