# Supplementary material for: Fabrication of heart tubes from iPSC derived cardiomyocytes and human fibrinogen by rotating mold technology
Source: Sci Rep. 2024 Jun 7;14:13174. doi: 10.1038/s41598-024-64022-7 (PMC11161509; doi:10.1038/s41598-024-64022-7)
Supplement: Supplementary file 1 — Supplementary Information 1. [file 41598_2024_64022_MOESM1_ESM.docx]

**Fabrication of heart tubes from iPSC derived cardiomyocytes and human fibrinogen by rotating mold technology**

Birgit Andrée^a^, Nils Voß^a^, Nils Kriedemann^a^, Wiebke Triebert^a^, Jana Teske^a^, Mira Mertens^a^, Merlin Witte^a^, Sára Szádocka^a^, Andres Hilfiker^a^, Thomas Aper^a^, Ina Gruh^a^, Robert Zweigerdt^a^

**
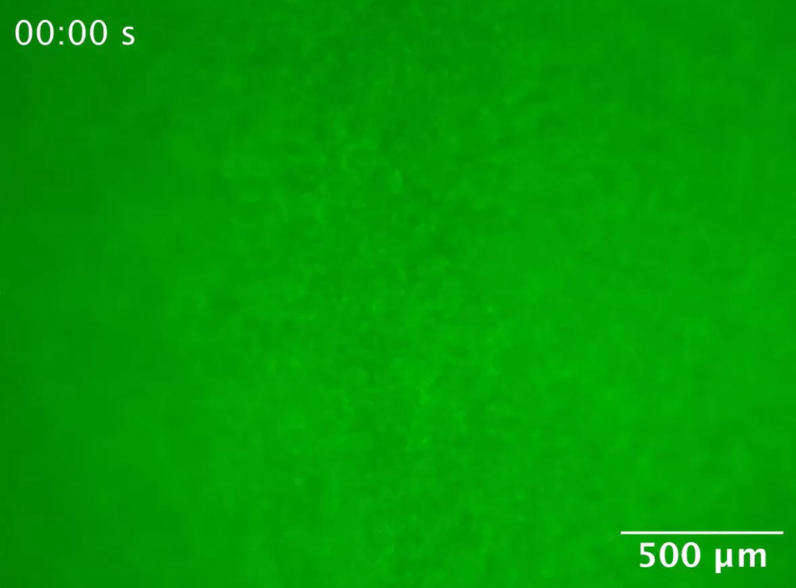
**

**Supplemental movie 1:**

**High cell density results in synchronized GCaMP6f signal.**

GCaMP6f signal recording of a RMT fibrin tube with 25x10^6^ cell/mL at day 7 of cultivation. Scale bar: 500 µm


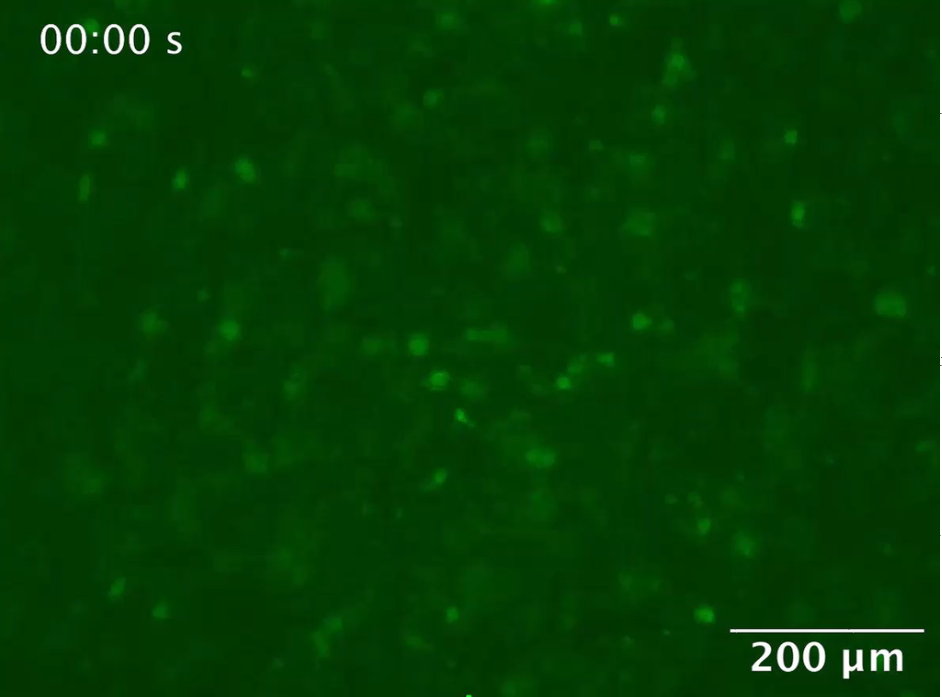


**Supplemental movie 2:**

**Static casting leads to isolated cells.**

GCaMP6f signal recording of static cast fibrin tube with 25x10^6^ cell/mL at day 8 of cultivation. Scale bar: 200 µm


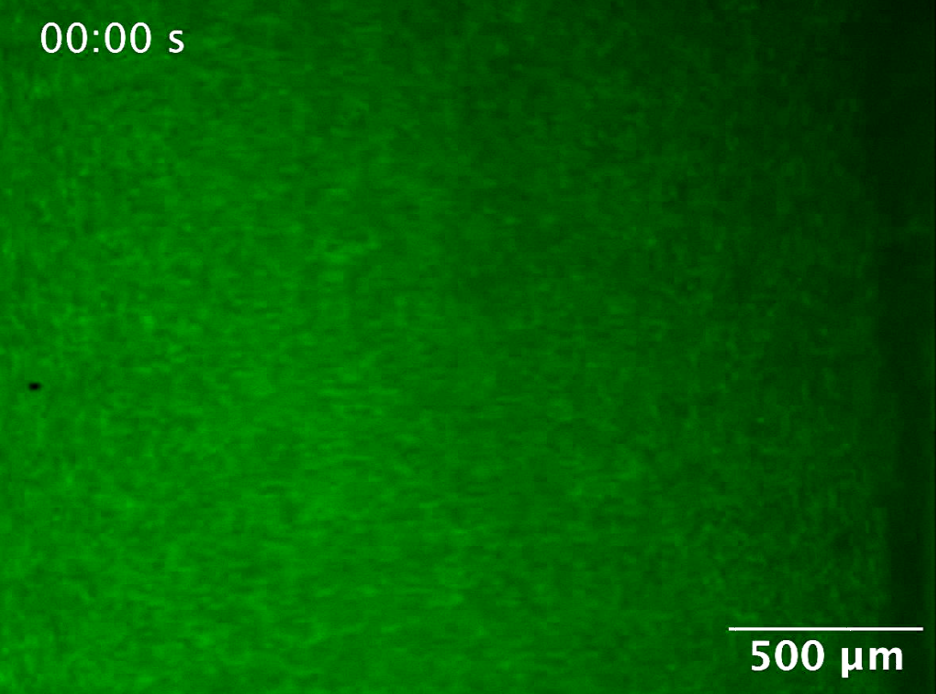


**Supplemental movie 3:**

RMT leads to synchronized Ca handling of CMs.

GCaMP6f signal recording of RMT fibrin tube with 25x10^6^ cell/mL at day 8 of cultivation. Scale bar: 500 µm


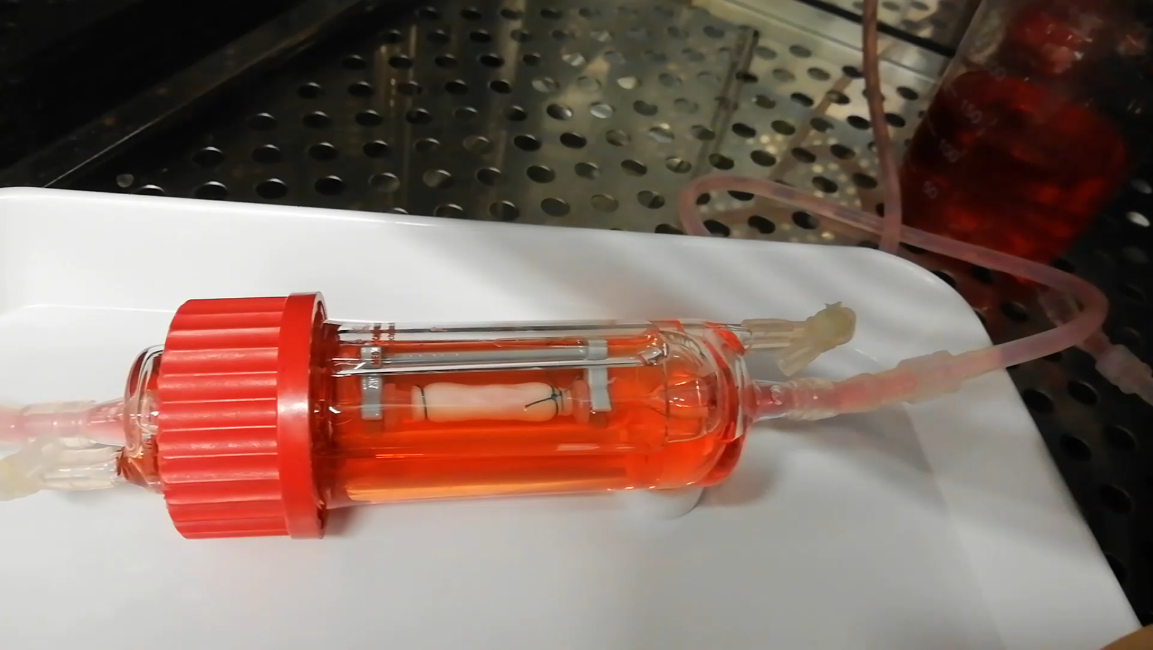


**Supplemental movie 4:**

**Bioreactor under pulsatile flow.**

RMT fibrin tube with sheath mounted in a bioreactor and cultivated under pulsatile flow with 10 mL/min and a frequency of 1 Hz.
